# Independent and Incremental Prognostic Value of the Endothelial Activation and Stress Index Beyond the GRACE Score for Predicting 1-Year Mortality in Patients with Non-ST-Segment Elevation Myocardial Infarction

**DOI:** 10.3390/medicina62071415

**Published:** 2026-07-21

**Authors:** Cagatay Onal, Burak Ayca

**Affiliations:** 1 Department of Cardiology, Private Gazi Hospital, Izmir 35230, Turkey; 2 Department of Cardiology, Buca Seyfi Demirsoy Training and Research Hospital, Izmir 35148, Turkey; drburakayca@yahoo.com.tr

**Keywords:** NSTEMI, EASIX, endothelial dysfunction, mortality, risk stratification, GRACE score

## Abstract

*Background and Objectives*: Risk stratification is an important tool for guiding clinical decision-making in patients with non-ST-segment elevation myocardial infarction (NSTEMI), yet mortality remains considerable despite contemporary therapeutic advances. Endothelial Activation and Stress Index (EASIX), calculated using lactate dehydrogenase, serum creatinine, and platelet count, is a readily available composite prognostic index that has demonstrated prognostic value across various cardiovascular settings. However, its role in NSTEMI has not been fully established. We therefore evaluated the association between EASIX and 1-year mortality and examined whether it provides incremental prognostic information beyond the GRACE risk score. *Materials and Methods*: We retrospectively evaluated 624 consecutive patients with NSTEMI who underwent invasive coronary angiography. EASIX was calculated from laboratory parameters obtained at admission. The prognostic significance of EASIX was evaluated using Cox proportional hazards models, Kaplan–Meier survival analysis, restricted cubic spline modelling, and incremental performance metrics. *Results*: During 1-year follow-up, 75 patients (12.0%) died. Admission EASIX values were higher among non-survivors than survivors (*p* < 0.001). Mortality increased progressively across EASIX tertiles (*p* < 0.001). In multivariable analyses, log_2_(EASIX) remained independently associated with mortality both in the clinical model (HR 1.381, *p* = 0.002) and after adjustment for the GRACE score (HR 1.315, *p* = 0.005). Restricted cubic spline analyses supported a graded relationship between EASIX and mortality risk. Addition of EASIX to the GRACE score improved discrimination (ΔAUC = 0.015, *p* = 0.032) and risk reclassification (continuous NRI = 0.385, *p* = 0.020). *Conclusions*: Admission EASIX emerged as an independent predictor of 1-year mortality among patients with NSTEMI. Furthermore, it provides incremental prognostic information beyond the GRACE risk score and may represent a simple, inexpensive, and readily available tool for risk stratification in contemporary NSTEMI practice.

## 1. Introduction

Despite advances in contemporary management, mortality and adverse cardiovascular outcomes remain substantial among patients with non-ST-segment elevation myocardial infarction (NSTEMI) [1]. Several factors have been identified as important determinants of prognosis in this population, including delayed hospitalization, reduced left ventricular ejection fraction, admission heart rate, and impaired hemodynamic status [2,3,4,5,6]. Accurate risk stratification therefore remains important for guiding clinical decision-making in patients with NSTEMI. The Global Registry of Acute Coronary Events (GRACE) score is currently the most established risk stratification tool for patients with NSTEMI. Numerous studies have demonstrated its value in predicting mortality and adverse cardiovascular outcomes, and recent large-scale validation studies have further confirmed its prognostic utility across different clinical settings and follow-up periods [7,8]. Nevertheless, biomarkers reflecting distinct pathophysiological pathways may offer additional prognostic insights in patients with NSTEMI.

Endothelial dysfunction and inflammation are increasingly recognized as important contributors to adverse cardiovascular outcomes in acute coronary syndromes [9]. The Endothelial Activation and Stress Index (EASIX), derived from lactate dehydrogenase, serum creatinine, and platelet count, was originally developed as a surrogate index associated with endothelial complications. However, its individual components are not specific markers of endothelial dysfunction, and EASIX may reflect multiple interrelated biological processes, including cellular injury, renal dysfunction, and platelet consumption. Emerging evidence supports the prognostic relevance of EASIX across a variety of cardiovascular conditions. Elevated EASIX levels have been associated with increased mortality risk in patients with coronary artery disease, chronic heart failure, and acute decompensated heart failure, highlighting its potential utility for cardiovascular risk stratification [10,11,12]. In a large population-based cohort, higher EASIX levels were associated with a greater burden of cardiovascular disease, including myocardial infarction, heart failure, angina, and stroke [13]. In addition, elevated EASIX levels have been independently associated with the no-reflow phenomenon in both STEMI and NSTEMI patients, further supporting its potential association with microvascular dysfunction in acute MI [14,15]. While the prognostic utility of EASIX has been explored in various cardiovascular settings, evidence in NSTEMI remains relatively limited. Accordingly, we sought to evaluate the association of EASIX with 1-year all-cause mortality in patients hospitalized with NSTEMI.

## 2. Materials and Methods

### 2.1. Study Population

**Figure 1:**
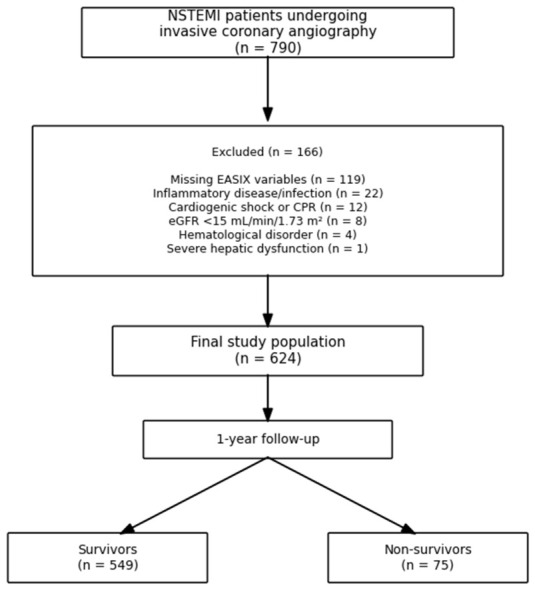
Study flowchart. Flow diagram showing patient screening, exclusion criteria, and the final study population included in the analysis.

We retrospectively evaluated consecutive patients admitted with NSTEMI who underwent coronary angiography at a tertiary care center between January 2019 and December 2024. Demographic, laboratory, clinical, and angiographic information were retrieved from the institutional electronic health record database. Patients were excluded if they had missing laboratory parameters required for calculation of the EASIX, end-stage renal disease, active malignancy, acute or chronic inflammatory disease, severe hepatic dysfunction, hematological disorders, cardiogenic shock at presentation, ongoing systemic infection, or a history of cardiac arrest requiring cardiopulmonary resuscitation before admission. Following exclusion of ineligible patients, a total of 624 individuals constituted the final analytical cohort. The patient selection process is summarized in Figure 1. This study evaluated an all-comer NSTEMI population treated according to contemporary guideline-directed practice. NSTEMI was diagnosed according to current ESC guideline recommendations [16].

### 2.2. Data Collection

Demographic data, cardiovascular risk factors, clinical characteristics at admission, laboratory measurements, angiographic findings, and discharge medications were obtained from the hospital’s electronic database. Blood samples were collected immediately after presentation to the emergency department and before coronary angiography. Hematological and biochemical parameters, including lactate dehydrogenase, serum creatinine, platelet count, cardiac troponin, and lipid profile, were measured using standard laboratory methods. Angiographic characteristics and treatment strategies were reviewed from procedural reports and medical records. No major systematic changes in institutional NSTEMI management protocols or laboratory measurement procedures occurred during the study period.

### 2.3. Calculation of EASIX

The admission EASIX score was calculated using laboratory parameters obtained at the time of hospital presentation according to the following formula: EASIX = lactate dehydrogenase (U/L) × serum creatinine (mg/dL)/platelet count (10^9^/L) [17].

### 2.4. Angiographic Assessment

Coronary angiography was performed in accordance with current clinical practice by experienced interventional cardiologists. Angiographic images and procedural reports were retrospectively reviewed to determine the culprit vessel, presence of left main coronary artery disease, and revascularization strategy. The extent and anatomical complexity of coronary artery disease were evaluated using the SYNTAX score. Subsequent management was categorized according to the initial treatment approach as PCI, CABG, or medical therapy alone.

### 2.5. Study Outcome

The primary study endpoint was 1-year all-cause mortality. Follow-up duration was calculated from the index hospitalization to death or completion of 1 year of follow-up. Mortality status was obtained from the national electronic health record system and official death registry databases. Patients were categorized as survivors or non-survivors according to their vital status at 1 year.

### 2.6. Statistical Analysis

Continuous variables are presented as median (interquartile range), and categorical variables as counts and percentages. Normality was assessed using the Kolmogorov–Smirnov test. No missing data were present for the variables included in the primary analyses; therefore, no imputation procedure was required. Continuous variables were compared using the Mann–Whitney U test or Kruskal–Wallis test, whereas categorical variables were analyzed using the chi-square test or Fisher’s exact test, as appropriate.

Because of its skewed distribution, EASIX was log-transformed using a base-2 logarithm [log_2_(EASIX)]. Hazard ratios were interpreted as the effect of a doubling increase in EASIX. Kaplan–Meier curves with log-rank testing were used for survival analyses according to both the optimal EASIX cut-off value determined by ROC analysis (Youden index) and EASIX tertiles.

Associations with 1-year all-cause mortality were evaluated using Cox proportional hazards regression. Variables with clinical relevance or significant univariable associations were entered into multivariable models. Model 1 included age, sex, body mass index, diabetes mellitus, left ventricular ejection fraction, Killip class ≥ II, SYNTAX score, and log_2_(EASIX), whereas Model 2 included the GRACE score and log_2_(EASIX). Given the number of outcome events relative to model complexity, the stability of Model 1 was further assessed by internal validation using 1000 bootstrap resamples. Model optimism was quantified, and optimism-corrected estimates of the C-index and calibration slope were obtained. The proportional hazards assumption was verified using Schoenfeld residuals, and multicollinearity was assessed using variance inflation factors (all VIFs < 2.0).

Restricted cubic spline (RCS) analyses were performed to explore potential non-linear associations between log_2_(EASIX) and mortality. Incremental prognostic value was assessed using ROC curve analysis with the DeLong test, continuous net reclassification improvement (NRI), and Harrell’s concordance index (C-index). Decision-curve analysis was performed for the 365-day mortality endpoint to assess the clinical utility of adding log_2_(EASIX) to the GRACE score by comparing the net benefit of the GRACE-only and combined models across threshold probabilities ranging from 2% to 30%. Model calibration was assessed by comparing predicted and observed 1-year mortality probabilities. Calibration curves were generated for the GRACE model and the GRACE plus log_2_(EASIX) model, and internal validation was performed using 1000 bootstrap resamples to estimate optimism-corrected calibration slopes. A calibration slope close to 1.0 was considered indicative of good agreement between predicted and observed risk.

Statistical analyses were performed using IBM SPSS Statistics version 23.0 and R version 4.4.1. A two-sided *p* value <0.05 was considered statistically significant.

## 3. Results

### 3.1. Patient Characteristics According to 1-Year Mortality

Among the 624 patients with NSTEMI included in the study, 75 (12.0%) died within 1 year. Baseline demographic, clinical, and laboratory characteristics according to 1-year survival status are summarized in Table 1. Relative to survivors, patients who died during follow-up were older and more frequently presented with diabetes mellitus and Killip class ≥ II. Non-survivors demonstrated lower body mass index and left ventricular ejection fraction, as well as a less favorable laboratory profile characterized by higher serum creatinine, lactate dehydrogenase, and neutrophil counts and lower lymphocyte counts. The type of revascularization strategy (PCI, CABG, or medical management) did not differ significantly between survivors and non-survivors (*p* = 0.471). However, non-survivors had significantly higher SYNTAX and GRACE scores (both *p* < 0.001). Furthermore, baseline EASIX values were significantly higher among non-survivors than among survivors [1.06 (0.73–1.79) vs. 0.71 (0.55–0.99), *p* < 0.001] (Table 1).

### 3.2. Patient Characteristics Across EASIX Tertiles

Clinical and laboratory characteristics according to admission EASIX tertiles are presented in Table 2 [Tertile 1 (T1) < 0.63; Tertile 2 (T2) 0.63–0.92; Tertile 3 (T3) > 0.92]. Patients in higher EASIX tertiles were significantly older and more frequently male. Increasing EASIX tertiles were associated with lower left ventricular ejection fraction, higher Killip class, higher creatinine and LDH levels, and lower lymphocyte and platelet counts (all *p* < 0.05).

Revascularization strategy differed significantly across EASIX tertiles (*p* < 0.001). The proportion of patients undergoing CABG increased progressively from 3.9% in T1 to 18.8% in T3. Similarly, SYNTAX scores were significantly higher in the highest tertile [T1: 9.00 vs. T2: 8.00 vs. T3: 14.00, *p* < 0.001]. Importantly, the incidence of 1-year mortality increased progressively across tertiles, from 4.3% in T1 to 10.1% in T2 and 21.6% in T3 (*p* < 0.001) (Table 2).

### 3.3. Cox Regression Analyses

In univariable Cox proportional hazards analyses, age, female sex, diabetes mellitus, lower body mass index, reduced left ventricular ejection fraction, higher creatinine levels, lower lymphocyte counts, higher SYNTAX scores, Killip class ≥ II, higher GRACE scores, and log_2_(EASIX) were related to an increased risk of 1-year mortality (Table 3). Each doubling of EASIX was associated with a 53.9% increase in mortality risk (HR 1.539, 95% CI 1.337–1.772, *p* < 0.001).

Following multivariable adjustment, log_2_(EASIX) remained a significant predictor of 1-year mortality in both models (Table 4). In Model 1, which included age, sex, body mass index, diabetes mellitus, left ventricular ejection fraction, Killip class ≥ II, and SYNTAX score, log_2_(EASIX) retained its prognostic significance (HR 1.381, 95% CI 1.129–1.689, *p* = 0.002). Age, lower body mass index, and Killip class ≥ II also remained independently associated with mortality. In Model 2, after adjustment for the GRACE score, log_2_(EASIX) retained its independent prognostic value (HR: 1.315, 95% CI: 1.087–1.591, *p* = 0.005). Model 1 included eight parameters and was based on 75 outcome events, corresponding to 9.4 events per parameter. In bootstrap internal validation with 1000 resamples, the apparent C-index of 0.835 was optimism-corrected to 0.819, while the optimism-corrected calibration slope was 0.900, supporting acceptable internal model stability. The proportional hazards assumption was satisfied for log_2_(EASIX) in both Model 1 (*p* = 0.664) and Model 2 (*p* = 0.580), based on Schoenfeld residual testing.

### 3.4. Kaplan–Meier Survival Analyses

**Figure 2:**
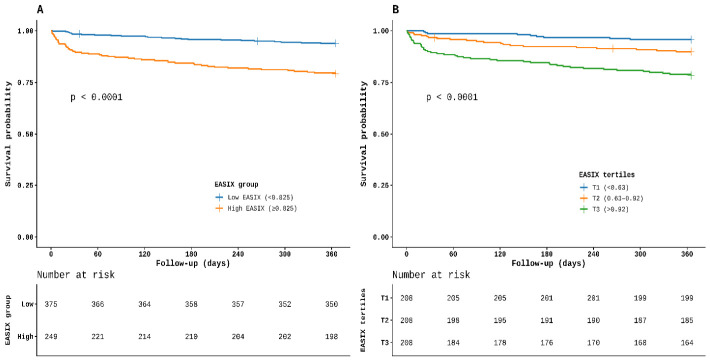
Kaplan–Meier analysis showing progressively lower survival rates as EASIX levels increase. (**A**) Survival according to the optimal EASIX cut-off value. (**B**) Survival according to EASIX tertiles.

The discriminative performance of EASIX for predicting 1-year mortality was evaluated using ROC analysis, yielding an AUC of 0.718. An optimal threshold of 0.825 was identified using the Youden criterion, providing a sensitivity of 76.7% and a specificity of 64.1%. Patients were subsequently stratified into lower- and higher-risk groups according to this cut-off value. Kaplan–Meier survival curves revealed a marked separation between the two groups, with patients exhibiting EASIX values ≥0.825 experiencing significantly poorer survival throughout follow-up (log-rank *p* < 0.0001; Figure 2A).

Similarly, survival decreased progressively across EASIX tertiles, with the highest mortality observed in T3 (>0.92), intermediate mortality in T2 (0.63–0.92), and the lowest mortality in T1 (<0.63) (log-rank *p* < 0.0001; Figure 2B).

### 3.5. Restricted Cubic Spline Analyses

**Figure 3:**
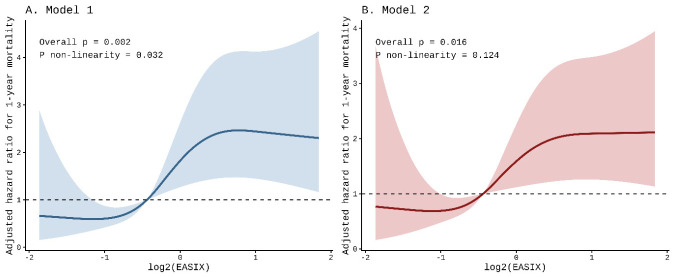
Restricted cubic spline analyses of the association between log_2_(EASIX) and 1-year all-cause mortality. (**A**) Model 1 adjusted for age, sex, body mass index, diabetes mellitus, left ventricular ejection fraction, Killip class ≥ II, and SYNTAX score. (**B**) Model 2 adjusted for the GRACE risk score. Solid lines represent adjusted hazard ratios, and shaded areas indicate 95% confidence intervals. The horizontal dashed line represents a hazard ratio of 1.0.

Restricted cubic spline analyses demonstrated a significant association between continuous log_2_(EASIX) levels and 1-year mortality in both adjustment models (Figure 3). In Model 1, the overall association was significant (Poverall = 0.002), with evidence of non-linearity (Pnon-linearity = 0.032). In Model 2, adjusted for the GRACE score, the overall association remained significant (Poverall = 0.016), whereas the non-linear component was no longer statistically significant (Pnon-linearity = 0.124).

### 3.6. Incremental Prognostic Value of EASIX

**Figure 4:**
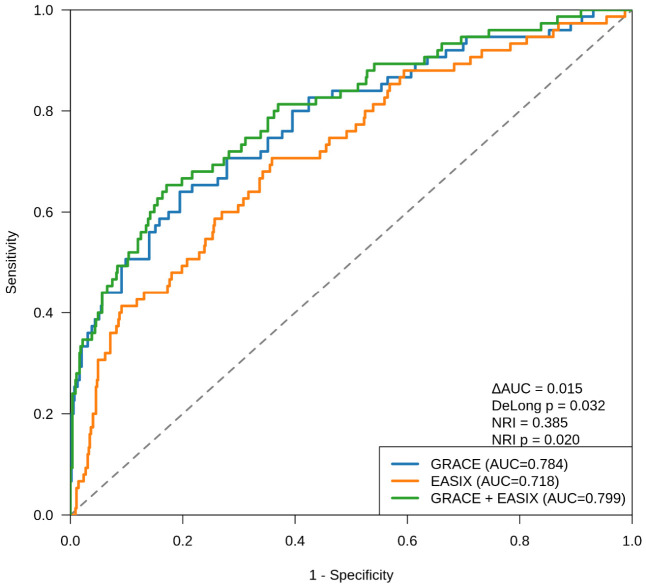
Incremental prognostic value of EASIX beyond the GRACE risk score for predicting 1-year mortality in patients with NSTEMI. Receiver operating characteristic (ROC) curves comparing the discriminatory performance of the GRACE score, EASIX, and the combined GRACE + EASIX model. The addition of EASIX to the GRACE score resulted in a significant improvement in predictive performance (ΔAUC = 0.015, DeLong *p* = 0.032). Net reclassification improvement (NRI) analysis also demonstrated a significant enhancement in risk stratification (NRI = 0.385, *p* = 0.020).

Among individual predictors, the GRACE score demonstrated the highest discrimination for 1-year mortality (AUC: 0.784, C-index: 0.773), followed by EASIX (AUC: 0.718, C-index: 0.708). The addition of EASIX to the GRACE model improved the AUC to 0.799, corresponding to a statistically significant increase in discrimination (ΔAUC = 0.015, DeLong *p* = 0.032; Figure 4). Furthermore, EASIX significantly improved risk reclassification, yielding a continuous NRI of 0.385 (95% CI: 0.004–0.673, *p* = 0.020). The combined model also achieved the highest C-index (0.787), supporting the incremental prognostic value of EASIX beyond the GRACE score (Table 5).

### 3.7. Model Calibration and Internal Validation

**Figure 5:**
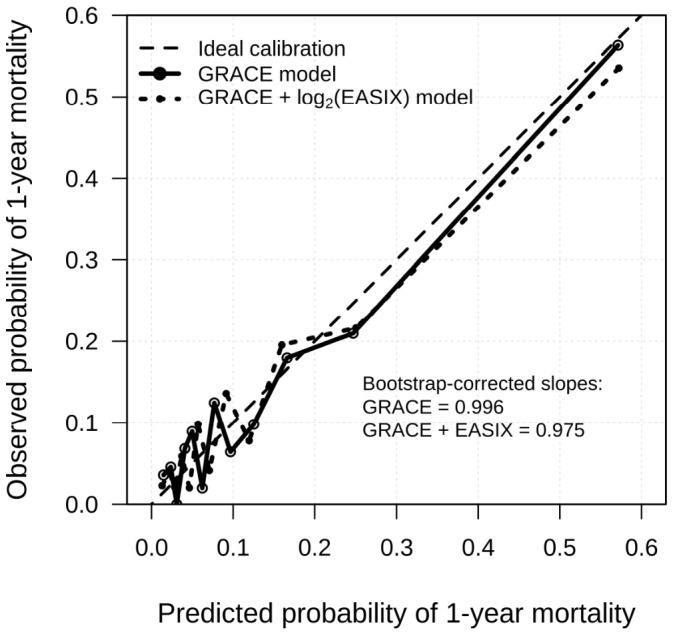
Calibration curves for the prediction of 1-year all-cause mortality using the GRACE model and the GRACE plus log_2_(EASIX) model. The dashed diagonal line represents ideal calibration. Model calibration was internally validated using 1000 bootstrap resamples. The optimism-corrected calibration slopes were 0.996 for the GRACE model and 0.975 for the GRACE plus log_2_(EASIX) model.

Calibration analysis demonstrated good agreement between predicted and observed 1-year mortality risks for both models. Following internal validation with 1000 bootstrap resamples, the optimism-corrected calibration slope was 0.996 for the GRACE model and 0.975 for the GRACE plus log_2_(EASIX) model. The calibration curves showed that predicted probabilities were generally consistent with observed event rates across the risk spectrum, supporting adequate calibration of both models (Figure 5).

### 3.8. Additional Analyses

The association between EASIX and mortality remained stable in additional analyses. Each 1-standard deviation increase in log_2_(EASIX) was linked to a 31.7% higher mortality risk in Model 1 and a 26.3% higher mortality risk in Model 2 (both *p* ≤ 0.005; Table 6). When evaluated categorically using the lowest tertile (T1) as the reference, patients in the highest tertile (T3) had a 3.60-fold higher mortality risk in Model 1 (HR: 3.597, 95% CI: 1.706–7.584, *p* < 0.001) and a 2.71-fold higher mortality risk after adjustment for the GRACE score (HR: 2.712, 95% CI: 1.300–5.659, *p* = 0.008). A significant linear trend across tertiles remained present in both models (*p* for trend = 0.003 for both).

### 3.9. Decision-Curve Analysis

**Figure 6:**
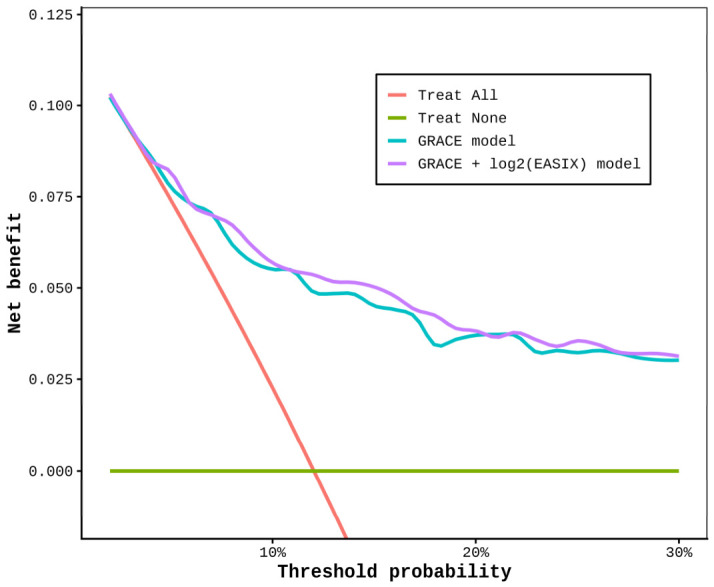
Decision-curve analysis comparing the net benefit of the GRACE score alone and the GRACE score combined with log_2_(EASIX) for predicting 1-year mortality.

Decision-curve analysis showed that the GRACE plus log_2_(EASIX) model provided a modestly greater net benefit than the GRACE-only model across threshold probabilities of approximately 5% to 20%. At higher threshold probabilities, the net benefits of the two models largely converged (Figure 6).

## 4. Discussion

Several important findings emerged from the present analysis. Higher EASIX levels were independently linked to an increased risk of 1-year mortality, with a progressive rise in risk observed across the EASIX spectrum. Furthermore, EASIX contributed prognostic information beyond that captured by the GRACE score. Collectively, these results underscore the potential utility of EASIX for risk assessment in patients with NSTEMI.

Accurate risk stratification remains important for guiding clinical decision-making in patients with NSTEMI, as clinical outcomes vary considerably among patients despite contemporary therapeutic advances. Consequently, several prognostic models have been developed to support therapeutic decision-making, among which the GRACE risk score remains the most extensively validated and guideline-recommended tool for mortality risk assessment and invasive strategy selection in patients with NSTEMI [7,16,18]. Nevertheless, contemporary data indicate that long-term mortality remains substantially elevated even among patients treated with PCI, particularly in those with a greater burden of comorbid conditions, highlighting the continued need for improved prognostic assessment in this population [19]. Consistent with these observations, advanced age, impaired renal function, reduced left ventricular ejection fraction, higher Killip class, and greater coronary artery disease complexity, as reflected by higher SYNTAX scores, were associated with mortality in our cohort and have been repeatedly linked to adverse outcomes in patients with NSTEMI and ACS populations [2,8,20,21,22,23,24]. In this context, our findings suggest that EASIX is independently associated with 1-year mortality and provides incremental prognostic information beyond the established GRACE risk score. The rationale for evaluating EASIX in addition to GRACE is that the GRACE score primarily incorporates clinical and hemodynamic variables. In contrast, EASIX may serve as an indirect composite surrogate integrating signals related to systemic cellular injury, renal dysfunction, platelet consumption or thrombo-inflammatory activation, and potentially microvascular or endothelial stress, which are not fully represented in conventional risk models.

EASIX was originally developed as a surrogate index associated with endothelial complications; however, its individual components are not specific markers of endothelial dysfunction [17]. Endothelial activation is increasingly recognized as a central mechanism underlying adverse cardiovascular outcomes through its effects on vascular inflammation, microvascular dysfunction, platelet activation, and impaired tissue perfusion. Circulating levels of soluble thrombomodulin, angiopoietin-2, CXCL8, CXCL9, and interleukin-18 have been documented to display substantial correlations with EASIX, thereby providing a plausible molecular rationale for its prognostic value [25,26,27]. These associations support the prognostic relevance of EASIX. However, rather than directly measuring endothelial dysfunction, EASIX may integrate systemic cellular injury, renal dysfunction, thrombo-inflammatory activation, and possible microvascular or endothelial stress.

Consistent with accumulating evidence linking endothelial dysfunction to adverse cardiovascular outcomes, our analysis identified log_2_(EASIX) as a significant determinant of 1-year mortality among patients presenting with NSTEMI. Similar findings have been reported in other cardiovascular settings. Finke et al. showed that increasing log_2_(EASIX) values were related to poorer long-term survival among individuals undergoing coronary angiography for coronary artery disease [10]. In a nationally representative population, Xia et al. demonstrated that higher EASIX values corresponded to greater risks of both all-cause and cardiovascular death and described a non-linear relationship between EASIX and adverse outcomes [28]. Likewise, Sang et al. reported that elevated EASIX values were linked to higher short-term mortality following acute MI, including NSTEMI presentations [29]. Additionally, while a previous study in NSTEMI patients undergoing PCI linked elevated EASIX levels exclusively to the acute occurrence of the no-reflow phenomenon [15], the present study extends these observations. Our findings demonstrate that the prognostic importance of EASIX in NSTEMI persists beyond the index hospitalization, extending from early microvascular complications to 1-year clinical mortality. A marked gradient in mortality risk was observed across EASIX tertiles, with 1-year mortality ranging from 4.3% in T1 to 21.6% in T3 (*p* < 0.001). Kaplan–Meier analyses likewise demonstrated significantly worse survival among patients with elevated EASIX levels. RCS analyses further confirmed a robust relationship between continuous log_2_(EASIX) values and mortality risk across both adjustment models (Poverall = 0.002 and 0.016, respectively). A significant nonlinear association was observed in the clinical model (Pnon-linearity = 0.032), whereas the relationship became more linear after adjustment for the GRACE risk score (Pnon-linearity = 0.124). The non-linear pattern observed in the clinical model may indicate that mortality risk increases disproportionately at higher EASIX levels, suggesting a potential threshold effect. However, the biological mechanisms underlying this association cannot be determined from the present analysis. Together, these findings support a graded relationship between increasing EASIX levels and mortality risk in patients with NSTEMI. Furthermore, the robustness of this association was supported by additional analyses. Patients in the highest EASIX tertile remained at substantially greater risk of death compared with those in the lowest tertile, even after adjustment for both clinical variables and the GRACE risk score. The persistence of a significant linear trend across tertiles further supports a dose–response relationship between increasing EASIX levels and mortality risk. Interestingly, higher EASIX levels were accompanied by higher SYNTAX scores and a greater frequency of CABG referral in our cohort. These findings suggest a potential association between EASIX and the anatomical complexity of coronary artery disease, although this relationship requires confirmation in future studies.

The prognostic relevance of EASIX has also been demonstrated across a wide range of cardiovascular disorders. Higher EASIX values have been linked to an increased risk of both all-cause and cardiovascular death among individuals with hypertension [30]. Similarly, among critically ill patients with heart failure, higher log_2_(EASIX) values were associated with increased 1-year mortality, while elevated EASIX levels also predicted acute kidney injury and in-hospital death [31,32]. In patients with STEMI undergoing primary PCI, EASIX has been shown to predict contrast-induced nephropathy, coronary no-reflow, and in-hospital mortality [33,34]. Collectively, these findings suggest that the prognostic utility of EASIX extends across diverse cardiovascular populations and clinical presentations, further supporting its biological and clinical relevance as a marker of adverse cardiovascular risk.

A notable observation of the present study was that EASIX improved risk prediction when incorporated into the GRACE risk score. To the best of our knowledge, previous cardiovascular studies evaluating EASIX have primarily focused on its independent prognostic performance, whereas data regarding its incremental value beyond established risk calculators remain limited. In our analysis, the addition of EASIX to the guideline-endorsed GRACE model resulted in a statistically significant improvement in discrimination (ΔAUC = 0.015, *p* = 0.032) and improved risk reclassification, as reflected by a continuous NRI of 0.385 (*p* = 0.020). These findings suggest that EASIX captures prognostic information not fully represented by conventional clinical variables. However, partial overlap between EASIX and the GRACE score should be acknowledged, as serum creatinine is incorporated into both measures. Therefore, part of the prognostic information captured by EASIX may reflect renal dysfunction. Nevertheless, the persistence of the association between log_2_(EASIX) and mortality after adjustment for the GRACE score suggests that its prognostic information may not be entirely attributable to renal function alone, with LDH and platelet count potentially providing complementary information. Although the improvement in discrimination was statistically significant, the absolute increase in AUC was modest and should therefore be interpreted cautiously. From a clinical perspective, EASIX should be regarded as a complementary rather than a competing tool to the GRACE score. Because it is derived from only three routinely available laboratory parameters, EASIX can be readily calculated without additional testing or cost and may provide insights into residual biological risk not fully captured by conventional clinical assessment. In this context, an elevated EASIX value may identify patients who could benefit from closer clinical surveillance and follow-up, including those with intermediate GRACE risk, in whom further risk refinement may be clinically relevant. However, the present findings do not establish EASIX-guided management thresholds or demonstrate that EASIX-based interventions improve outcomes. Therefore, prospective external validation and dedicated impact studies are required before routine clinical implementation.

### Limitations

Several limitations of the present study should be acknowledged. First, this was a retrospective single-center study, and therefore, the possibility of residual confounding cannot be completely excluded despite multivariable adjustment. Second, EASIX was calculated from a single set of laboratory measurements obtained at admission, and serial changes in EASIX during hospitalization were not evaluated. Third, although we adjusted for established clinical and angiographic risk factors, information regarding some potentially relevant variables, including inflammatory biomarkers, frailty status, and longitudinal treatment adherence, was not available. Fourth, the study population was limited to patients with NSTEMI who underwent invasive coronary angiography at a tertiary referral center, which may restrict the generalizability of the findings to broader NSTEMI populations and other healthcare settings. Fifth, the present study evaluated only all-cause mortality as the primary outcome. Information on other clinically relevant endpoints, including major adverse cardiovascular events, repeat revascularization, and cardiovascular mortality, was not consistently available and therefore could not be assessed. Finally, the observational design precludes causal inference, and prospective multicenter studies are required to validate the prognostic utility of EASIX and determine whether EASIX-guided risk stratification can improve clinical outcomes. Although internal validation using bootstrap resampling supported model stability, this approach cannot substitute for external validation. Therefore, the prognostic and incremental value of EASIX should be confirmed in independent, prospective, multicenter cohorts before its routine clinical implementation can be recommended.

## 5. Conclusions

In this cohort of patients with NSTEMI, higher EASIX levels were independently associated with increased 1-year all-cause mortality. This relationship remained robust across multiple complementary analytical approaches, including multivariable Cox regression, Kaplan–Meier survival analyses, RCS modeling, and risk stratification according to EASIX tertiles. Furthermore, EASIX provided incremental prognostic information beyond the established GRACE risk score, improving both discrimination and risk reclassification. Given its simplicity, low cost, and widespread availability, EASIX may represent a useful adjunctive tool for the assessment of 1-year all-cause mortality risk in contemporary NSTEMI practice. Larger prospective multicenter investigations are needed to confirm these findings and to explore the association of EASIX with other clinically relevant outcomes, including major adverse cardiovascular events.

## Figures and Tables

**Table 1:** Baseline Characteristics According to 1-Year Mortality Status.

Variable	Survivors (*n* = 549)	Non-Survivors (*n* = 75)	*p* Value
Demographic and clinical characteristics			
Age, years	61.00 (53.00–70.00)	76.00 (64.00–81.00)	<0.001
Male sex, *n* (%)	404 (73.6)	47 (62.7)	0.065
Body mass index, kg/m^2^	28.01 (24.91–31.22)	25.39 (23.44–29.38)	0.001
Smoking status, *n* (%)	321 (58.5)	38 (50.7)	0.247
Hypertension, *n* (%)	284 (51.7)	34 (45.3)	0.359
Diabetes mellitus, *n* (%)	186 (33.9)	35 (46.7)	0.041
Hemodynamic and clinical presentation			
Systolic blood pressure, mmHg	135.00 (116.00–154.00)	132.00 (106.00–155.00)	0.327
Heart rate, bpm	77.00 (65.00–89.00)	99.00 (83.00–109.00)	<0.001
Left ventricular ejection fraction, %	60.00 (50.00–60.00)	50.00 (40.00–60.00)	<0.001
Killip class, *n* (%)			<0.001
I	535 (97.4)	51 (68.0)	
II	12 (2.2)	14 (18.7)	
III	2 (0.4)	10 (13.3)	
Laboratory findings			
Creatinine, mg/dL	0.84 (0.70–1.00)	1.00 (0.89–1.40)	<0.001
Lactate dehydrogenase, U/L	208.80 (191.50–226.10)	252.70 (233.50–276.00)	<0.001
Neutrophil count, ×10^3^/µL	5.53 (4.28–7.06)	6.55 (4.69–8.08)	0.009
Lymphocyte count, ×10^3^/µL	2.28 (1.70–2.90)	1.39 (1.03–2.13)	<0.001
Platelet count, ×10^3^/µL	246.00 (202.00–296.00)	244.00 (203.50–312.00)	0.885
LDL-C, mg/dL	125.00 (93.00–157.00)	116.00 (86.00–138.50)	0.016
HDL-C, mg/dL	41.00 (35.00–47.00)	38.00 (31.00–44.00)	0.018
Triglycerides, mg/dL	154.00 (115.00–213.00)	111.00 (87.50–174.50)	<0.001
EASIX score	0.71 (0.55–0.99)	1.06 (0.73–1.79)	<0.001
Angiographic characteristics			
Culprit vessel, *n* (%)			0.436
LAD	243 (44.3)	28 (37.3)	
CX	144 (26.2)	24 (32.0)	
RCA	133 (24.2)	16 (21.3)	
LMCA	19 (3.5)	5 (6.7)	
Others	10 (1.8)	2 (2.7)	
LMCA disease, *n* (%)	19 (3.5)	4 (5.6)	0.337
Revascularization strategy, *n* (%)			0.471
PCI	493 (89.8)	64 (85.3)	
CABG	49 (8.9)	10 (13.3)	
Medical treatment	7 (1.3)	1 (1.3)	
SYNTAX score	9.00 (6.00–15.00)	15.00 (10.00–24.00)	<0.001
GRACE score	118.00 (101.00–139.00)	158.00 (129.00–187.50)	<0.001
Discharge medications			
ACEI/ARB, *n* (%)	447 (81.4)	55 (73.3)	0.133
Beta-blocker, *n* (%)	470 (85.6)	64 (85.3)	1.000
Statin, *n* (%)	219 (39.9)	42 (56.0)	0.011
Aspirin, *n* (%)	479 (87.2)	67 (89.3)	0.745
P2Y12 inhibitor, *n* (%)	500 (91.1)	64 (85.3)	0.170

Data are presented as median (interquartile range) or *n* (%). Abbreviations: ACEI, angiotensin-converting enzyme inhibitor; ARB, angiotensin receptor blocker; BMI, body mass index; EASIX, Endothelial Activation and Stress Index; GRACE, Global Registry of Acute Coronary Events; HDL-C, high-density lipoprotein cholesterol; LDL-C, low-density lipoprotein cholesterol; LMCA, left main coronary artery.

**Table 2:** Baseline Characteristics According to EASIX Tertiles.

Variable	T1 (<0.63) (*n* = 208)	T2 (0.63–0.92) (*n* = 208)	T3 (>0.92) (*n* = 208)	*p* Value
Demographic and clinical characteristics				
Age, years	58.00 (50.00–66.00)	61.00 (54.00–72.00)	67.00 (58.75–75.00)	<0.001
Male sex, *n* (%)	138 (66.3)	147 (70.7)	166 (79.8)	0.007
Body mass index, kg/m^2^	27.14 (24.11–31.05)	28.33 (25.38–31.16)	27.92 (24.79–31.14)	0.045
Smoking status, *n* (%)	130 (62.5)	115 (55.3)	114 (54.8)	0.206
Hypertension, *n* (%)	108 (51.9)	109 (52.4)	101 (48.6)	0.694
Diabetes mellitus, *n* (%)	68 (32.7)	76 (36.5)	77 (37.0)	0.600
Hemodynamic and clinical presentation				
Systolic blood pressure, mmHg	135.00 (115.00–153.25)	134.50 (115.00–154.00)	135.00 (115.00–155.00)	0.978
Heart rate, bpm	79.00 (65.75–91.00)	77.50 (65.75–90.25)	80.00 (67.00–94.00)	0.253
Left ventricular ejection fraction, %	60.00 (55.00–60.00)	60.00 (50.00–60.00)	55.00 (47.25–60.00)	<0.001
Killip class, *n* (%)				0.002
I	203 (97.6)	199 (95.7)	184 (88.5)	
II	3 (1.4)	6 (2.9)	17 (8.2)	
III	2 (1.0)	3 (1.4)	7 (3.4)	
Laboratory findings				
Creatinine, mg/dL	0.70 (0.64–0.80)	0.86 (0.76–0.98)	1.04 (0.90–1.35)	<0.001
Lactate dehydrogenase, U/L	201.05 (183.70–217.03)	210.65 (193.20–227.32)	226.50 (206.78–257.52)	<0.001
Neutrophil count, ×10^3^/µL	5.83 (4.76–7.77)	5.54 (4.19–7.15)	5.48 (4.04–7.15)	0.017
Lymphocyte count, ×10^3^/µL	2.46 (1.79–3.13)	2.30 (1.74–2.90)	1.87 (1.23–2.41)	<0.001
Platelet count, ×10^3^/µL	301.00 (263.00–350.25)	242.50 (210.00–282.25)	197.00 (164.00–232.25)	<0.001
LDL-C, mg/dL	124.50 (93.75–160.00)	123.50 (91.00–153.00)	123.00 (88.00–153.25)	0.361
HDL-C, mg/dL	42.00 (35.00–48.00)	41.00 (35.00–46.00)	38.50 (33.00–44.00)	0.001
Triglycerides, mg/dL	150.00 (108.75–200.00)	157.50 (115.00–224.25)	142.50 (105.50–189.75)	0.124
Angiographic and risk characteristics				
Culprit vessel, *n* (%)				0.306
LAD	98 (47.1)	80 (38.5)	93 (44.7)	
CX	50 (24.0)	59 (28.4)	59 (28.4)	
RCA	51 (24.5)	58 (27.9)	40 (19.2)	
LMCA	5 (2.4)	7 (3.4)	12 (5.8)	
Others	4 (1.9)	4 (1.9)	4 (1.9)	
LMCA disease, *n* (%)	8 (3.9)	6 (2.9)	9 (4.6)	0.675
Revascularization strategy, *n* (%)				<0.001
PCI	194 (94.2)	197 (93.4)	166 (80.2)	
CABG	8 (3.9)	12 (5.7)	39 (18.8)	
Medical treatment	4 (1.9)	2 (0.9)	2 (1.0)	
SYNTAX score	9.00 (6.00–13.50)	8.00 (5.00–15.00)	14.00 (9.00–18.62)	<0.001
GRACE score	112.50 (95.00–130.00)	118.00 (96.00–141.00)	136.00 (113.50–159.00)	<0.001
1-year mortality, *n* (%)	9 (4.3)	21 (10.1)	45 (21.6)	<0.001

Data are presented as median (interquartile range) or *n* (%). Abbreviations: BMI, body mass index; EASIX, Endothelial Activation and Stress Index; GRACE, Global Registry of Acute Coronary Events; HDL-C, high-density lipoprotein cholesterol; LDL-C, low-density lipoprotein cholesterol; LMCA, left main coronary artery.

**Table 3:** Univariable Cox Regression Analysis for 1-Year Mortality.

Variable	HR	95% CI	*p* Value
Age	1.077	1.056–1.099	<0.001
Female sex	1.635	1.024–2.610	0.040
Body mass index	0.926	0.88–0.974	0.003
Diabetes mellitus	1.652	1.049–2.600	0.030
Left Ventricular Ejection Fraction	0.945	0.926–0.964	<0.001
Creatinine	2.256	1.694–3.004	<0.001
Neutrophil count	0.997	0.985–1.010	0.685
Lymphocyte count	0.453	0.338–0.606	<0.001
SYNTAX score	1.066	1.046–1.085	<0.001
GRACE score	1.035	1.029–1.042	<0.001
Killip class ≥ II	12.600	7.726–20.551	<0.001
log_2_(EASIX)	1.539	1.337–1.772	<0.001

HRs are presented with 95% confidence intervals. Abbreviations: BMI, body mass index; CI, confidence interval; EASIX, Endothelial Activation and Stress Index; GRACE, Global Registry of Acute Coronary Events; HR, hazard ratio.

**Table 4:** Multivariable Cox Regression Analysis for 1-Year Mortality.

Variable	HR	95% CI	*p* Value
**Model 1**			
Age	1.052	1.029–1.076	<0.001
Female sex	1.068	0.616–1.851	0.816
Body mass index	0.935	0.891–0.981	0.006
Diabetes mellitus	1.393	0.861–2.253	0.176
Left Ventricular Ejection Fraction	0.996	0.969–1.023	0.754
Killip class ≥ II	5.647	2.926–10.898	<0.001
SYNTAX score	1.013	0.986–1.040	0.354
log_2_(EASIX)	1.381	1.129–1.689	0.002
**Model 2**			
GRACE score	1.033	1.027–1.040	<0.001
log_2_(EASIX)	1.315	1.087–1.591	0.005

Model 1 included age, female sex, body mass index, diabetes mellitus, left ventricular ejection fraction, Killip class ≥ II, SYNTAX score, and log_2_(EASIX). Model 2 included GRACE score and log_2_(EASIX). The proportional hazards assumption was evaluated using Schoenfeld residuals. For the primary predictor, log_2_(EASIX), no evidence of violation of the proportional hazards assumption was observed in either Model 1 (*p* = 0.664) or Model 2 (*p* = 0.580). The global proportional hazards assumption was satisfied for Model 2 (global test *p* = 0.29). Abbreviations: BMI, body mass index; CI, confidence interval; EASIX, Endothelial Activation and Stress Index; GRACE, Global Registry of Acute Coronary Events; HR, hazard ratio.

**Table 5:** Incremental Predictive Value of EASIX Added to the GRACE Score.

Model/Comparison	AUC/Estimate	95% CI	C-Index	*p* Value
GRACE	0.784	0.723–0.844	0.773	
EASIX	0.718	0.653–0.782	0.708	
GRACE + EASIX	0.799	0.741–0.857	0.787	
GRACE vs. GRACE + EASIX	ΔAUC = 0.015			0.032
EASIX vs. GRACE + EASIX	ΔAUC = 0.081			0.016
GRACE vs. EASIX	ΔAUC = 0.066			0.090
Continuous NRI	0.385	0.004–0.673		0.020

Pairwise comparisons between ROC curves were performed using the DeLong test. Continuous net reclassification improvement (NRI) was calculated to evaluate the incremental prognostic value of adding EASIX to the GRACE score. Abbreviations: AUC, area under the curve; CI, confidence interval; C-index, concordance index; EASIX, Endothelial Activation and Stress Index; GRACE, Global Registry of Acute Coronary Events; NRI, net reclassification improvement.

**Table 6:** Additional analyses of the association between EASIX and 1-year mortality.

Variable	Model 1 HR (95% CI)	Model 1 *p* Value	Model 2 HR (95% CI)	Model 2 *p* Value
log_2_(EASIX) per 1-SD increase	1.317 (1.109–1.563)	0.002	1.263 (1.074–1.485)	0.005
T1 (lowest tertile)	Reference		Reference	
T2 vs. T1	2.071 (0.931–4.606)	0.074	1.644 (0.745–3.628)	0.219
T3 vs. T1	3.597 (1.706–7.584)	<0.001	2.712 (1.300–5.659)	0.008
P for trend (per tertile increase)	1.857 (1.323–2.607)	<0.001	1.648 (1.183–2.296)	0.003

Model 1 was adjusted for age, sex, body mass index, diabetes mellitus, left ventricular ejection fraction, Killip class ≥ II, and SYNTAX score. Model 2 was adjusted for the GRACE score. P for trend was calculated by entering EASIX tertiles as an ordinal variable into the multivariable Cox proportional hazards model. Tertile cut-off values were defined as T1 < 0.63, T2 = 0.63–0.92, and T3 > 0.92. Abbreviations: CI, confidence interval; EASIX, Endothelial Activation and Stress Index; GRACE, Global Registry of Acute Coronary Events; HR, hazard ratio; SD, standard deviation; SYNTAX, Synergy Between Percutaneous Coronary Intervention With Taxus and Cardiac Surgery.

## Data Availability

The datasets generated and/or analyzed during the current study are not publicly available due to institutional policies and patient confidentiality considerations but are available from the corresponding author on reasonable request.
